# Food Consumption Data as a Tool to Estimate Exposure to Mycoestrogens

**DOI:** 10.3390/toxins12020118

**Published:** 2020-02-13

**Authors:** Carla Martins, Duarte Torres, Carla Lopes, Daniela Correia, Ana Goios, Ricardo Assunção, Paula Alvito, Arnau Vidal, Marthe De Boevre, Sarah De Saeger, Carla Nunes

**Affiliations:** 1 Food and Nutrition Department, National Institute of Health Dr. Ricardo Jorge, Avenida Padre Cruz, 1649-016 Lisboa, Portugal; ricardo.assuncao@insa.min-saude.pt (R.A.); paula.alvito@insa.min-saude.pt (P.A.); 2 CESAM, Centre for Environmental and Marine Studies, University of Aveiro, Campus Universitário de Santiago, 3810-193 Aveiro, Portugal; 3 NOVA National School of Public Health, Public Health Research Centre, Universidade NOVA de Lisboa, Avenida Padre Cruz, 1600-560 Lisboa, Portugal; cnunes@ensp.unl.pt; 4 Comprehensive Health Research Center (CHRC), Universidade NOVA de Lisboa, Campo Mártires da Pátria, 1169-056 Lisboa, Portugal; 5 Faculty of Nutrition and Food Sciences, University of Porto, Rua Dr. Roberto Frias, 4200-465 Porto, Portugal; dupamato@fcna.up.pt (D.T.); aclgoios@gmail.com (A.G.); 6 Epidemiology Research Unit, Institute of Public Health, University of Porto, Rua das Taipas 135, 4050-091 Porto, Portugal; carlal@med.up.pt (C.L.); danielamc@med.up.pt (D.C.); 7 Department of Public Health and Forensic Sciences, and Medical Education, Faculty of Medicine, University of Porto, Alameda Prof. Hernâni Monteiro, 4200-319 Porto, Portugal; 8 Centre of Excellence in Mycotoxicology and Public Health, Faculty of Pharmaceutical Sciences, Ghent University, Ottergemsesteenweg 460, B-9000 Ghent, Belgium; arnau.vidalcorominas@ugent.be (A.V.); Marthe.DeBoevre@UGent.be (M.D.B.); Sarah.DeSaeger@UGent.be (S.D.S.)

**Keywords:** modelling, mycotoxins, food consumption, urinary biomarkers, public health

## Abstract

Zearalenone and alternariol are mycotoxins produced by *Fusarium* and *Alternaria* species, respectively, that present estrogenic activity and consequently are classified as endocrine disruptors. To estimate the exposure of the Portuguese population to these two mycotoxins at a national level, a modelling approach, based on data from 94 Portuguese volunteers, was developed considering as inputs: i) the food consumption data generated within the National Food and Physical Activity Survey; and ii) the human biomonitoring data used to assess the exposure to the referred mycotoxins. Six models of association between mycoestrogens urinary levels (zearalenone, total zearalenone and alternariol) and food items (meat, cheese, and fresh-cheese, breakfast cereals, sweets) were established. Applying the obtained models to the consumption data (*n* = 5811) of the general population, the median estimates of the probable daily intake revealed that a fraction of the Portuguese population might exceed the tolerable daily intake defined for zearalenone. A reference intake value for alternariol is still lacking, thus the characterization of risk due to the exposure to this mycotoxin was not possible to perform. Although the unavoidable uncertainties, these results are important contributions to understand the exposure to endocrine disruptors in Portugal and the potential Public Health consequences.

## 1. Introduction

Mycotoxins are secondary metabolites produced by filamentous fungi and represent one of the most relevant group of food contaminants [1]. These toxins are considered ubiquitous and can affect the food chain in the different stages of production, harvest, storage, and processing [2]. They are associated with several health outcomes in humans and animals, including carcinogenic, immunotoxic, nephrotoxic, neurotoxic, teratogenic, and hepatotoxic effects [1,3]. Some mycotoxins, such as zearalenone (ZEN) and alternariol (AOH) produced by *Fusarium* and *Alternaria* species respectively, present estrogenic activity and are classified as endocrine disruptors [4,5,6]. According to the World Health Organization (WHO), an endocrine disruptor is a “substance that alter one or more function of the endocrine system and consequently cause adverse effects in an intact organism, its progeny and a (sub)population” [7]. Endocrine disruptors interfere with the hormones’ action, disrupt homeostasis, and may alter physiology during the whole life span of an individual, from foetal development to adult growth [8].

As referred by several authors, ZEN presents a chemical structure that resembles the structure of naturally-occurring estrogens, namely 17-β-estradiol, making ZEN capable of binding to estrogen receptors (full agonist to ER-α, mixed agonist–antagonist to ER-β) [9,10,11]. ZEN has been implicated in the disruption of mammalian reproduction by affecting the synthesis and secretion of sex hormones such as progesterone, estradiol, and testosterone [12]. It was also reported as presenting a higher estrogenic relative potency factor than bisphenol A (BPA) [13,14]. ZEN is characterized by a fast metabolism, within 24 hours, and the excretion rates reported so far are 9.4% obtained in a human intervention study, and 36.8% obtained in an animal study [15,16]. The metabolism comprehends a phase I of reduction reactions, and a phase II of glucuronidation/sulfonation reactions [17]. Epidemiological studies also revealed the presence of zearalenone and metabolites in biological samples, confirming that human exposure to this mycotoxin is commonplace [18,19,20,21,22,23]. The consequences of human exposure to ZEN were suggested by several results. In Hungary [24], Italy [25], and Turkey [26], regions where increasing cases of early telarche and central idiopathic precocious puberty were reported, an association with ZEN and metabolites levels in biological samples was established. The latest results of Jersey Girls Study showed that girls with detectable mycoestrogen levels were significantly shorter in stature at menarche compared to girls with undetectable levels [27]. The occurrence of ZEN is mainly reported in cereals and animal products, and the ingestion of these food commodities is considered the major source of human exposure [28]. In the Regulation 1881/2006 and its amendments, the European Commission established maximum admissible levels for the occurrence of ZEN in foods. These levels were laid down for cereals and cereal-products such as bread (including small bakery wares), pastries, biscuits, cereal snacks and breakfast cereals, and cereal-based foods intended for infants and young children consumption, ranging between 20 and 400 µg kg^-1^ [29]. Based on the estrogenic effects of ZEN in pigs as the critical endpoint, the European Food Safety Authority (EFSA) recommended a tolerable daily intake (TDI) for ZEN and metabolites of 250 ng kg^-1^ bw day^-1^ [28]. 

Regarding AOH, very little data concerning metabolism and toxicity is available. Alternariol has been associated with genotoxic and mutagenic effects and it is considered an endocrine disruptor, being also capable of binding to estrogens’ receptors (ER-β, preferentially) [30]. Recently, in a study developed by Puntscher et al. in rats, it was possible to determine an excretion rate of 8.3% for AOH, with an increase of 7% and 19% when urine and faeces samples were pre-treated with β-glucuronidase/arylsulphatase, indicating that glucuronidation and sulfonation are potentially metabolic pathways also for AOH [30]. Alternariol was recently identified and quantified in urine samples [19,31], confirming the human exposure to this mycotoxin. *Alternaria* toxins are considered important emerging risks that need to be properly assessed in food safety, yet there are no regulations for the toxins in food and feed in Europe [32]. The occurrence of AOH has been reported in fruits, vegetables and vegetable-products, cereals and cereal-products, dried fruits and nuts, sunflower seeds, wines, and infant foods [33,34,35]. The last assessment conducted by EFSA in 2016 concerning *Alternaria* toxins concluded there is a need for more sensitive analytical methods in order to decrease the amount of left-censored data and the uncertainty associated with risk assessment [36]. In the absence of a TDI for *Alternaria* toxins, EFSA applied the threshold for toxicological concern (TTC) approach to characterize the risk and concluded that exposure to AOH and alternariol monomethyl ether (AME) could represent a health concern [36].

Regarding the endocrine disruptive activity, the interaction between ZEN and AOH was assessed through models using Ishikawa cells and synergistic effects were observed [9].

Since food intake is considered the major source of exposure to mycotoxins, it is also important to shed a light on the food commodities that may be the determinants of this exposure. Several studies attempted to assess the association between food intake and mycotoxins’ urinary biomarkers, with the majority of the studies using bivariate analysis (correlation coefficients). None reported associations for *Alternaria* toxins, namely AOH, and only one reported an association of grains and meat intake with exposure to ZEN [21]. Recently, Mitropoulou et al. [37] found a weak association between urinary ZEN concentrations and chocolate consumption using bivariate analysis, but no association were found when performing a more extensive statistical analysis and using a multivariate median regression analysis. From our knowledge, the only study thus far that has performed a deep statistical analysis to assess the influence of food consumption in mycotoxins’ urinary biomarkers was performed by Turner et al. [38]. This study intended to find significant contributors for the exposure to deoxynivalenol in the UK population. The authors gathered data from urinary excretion of mycotoxins obtained from 24 h urine samples as well as food consumption data from the National Data and Nutrition Survey, and through a multivariate model reported wholemeal bread, white bread, buns/cakes, high fibre breakfast cereals, and pasta as significant contributors for exposure to deoxynivalenol [38]. Martins et al. [39] note that for mycotoxins with short half-lives, such as ZEN and AOH, it is expected to find associations between food consumption of the previous 24 h and urinary biomarkers of exposure.

A recent human biomonitoring study developed in Portugal revealed the presence of mycoestrogens in 24 h urine and first morning urine of 94 volunteers [19]. Regarding ZEN, estimates revealed that 24% of participants would surpass the established TDI [19]. Data to properly characterize the risk associated to AOH exposure were not available. These obtained results contributed to the recognition that the Portuguese population are exposed to mycoestrogens. In order to establish preventive public health measures and to anticipate potential health effects, a deeper analysis of exposure scenarios for the different age groups, regions, and sex is of utmost importance. The identification of the main food contributors through association with food consumption data could be further explored for the development of modelling tools to estimate exposure to a larger and more representative sample.

Considering the above, the recently obtained food consumption data under the National Food and Physical Activity Survey (IAN-AF) were combined with the data regarding human exposure to mycotoxins obtained through a human biomonitoring (HBM) study [19,40] aiming to: i) to develop a statistical model relating food consumption and mycotoxins exposure; and ii) to estimate the exposure to ZEN and AOH of all the participants of IAN-AF, stratified by age, sex, and region, based on the developed modelling.

## 2. Results and Discussion

### 2.1. HBM Data

#### 2.1.1. Sociodemographic Characterization of Participants

The sociodemographic characteristics of participants are described in Table 1. Participants in the HBM study (*n* = 94) were similarly distributed by sex, with 51.1% of males and 48.9% of females, and were mainly from the northern region of Portugal (78.7%). Regarding the educational level, about half of the participants (51.1%) reported 9 years or less of education. Only 13.8% reported a monthly income above 1941€ and 55.3% of the participants were workers for remuneration or profit.

Participants in the IAN-AF study (*n* = 5811) were similarly distributed by sex, with 48.1% of males and 51.9% of females, and presented a distribution across the country with similar percentages from all regions. This group included participants from all age groups. Regarding the educational level, 44.5% of the participants reported 10–12 years of education. Regarding the monthly income, the range of 485–1455 € was reported by almost half of the participants (49.0%). More than half of the participants (55%) were workers for remuneration or profit.

Both groups of participants presented similar sociodemographic characteristics. The group of 5811 participants was representative of the Portuguese population at regional level.

#### 2.1.2. Mycoestrogens’ Urinary Biomarkers

Martins et al. [19] reported results for the urinary biomarkers of mycoestrogens in a human biomonitoring study where 24 h urine and first morning urine (FMU) paired samples of participants from north and centre regions of Portugal were analysed. Regarding 24 h urine samples, the authors reported positive samples above the limit of detection (LOD) for ZEN (48%), ZEN-14-GlcA (16%), and AOH (29%) [19]. Regarding FMU samples, the authors reported positive samples (>LOD) for ZEN (57%), ZEN-14-GlcA (16%), α-ZEL (5%), and AOH (13%) [19]. Other known metabolites of ZEN, namely beta-zearalenol (β-ZEL), alpha-zearalanol (α-ZAL), beta-zearalanol (β-ZAL), alpha-zearalenol-glucuronide (α-ZEL-GlcA), beta-zearalenol-glucuronide (β-ZEL-GlcA), and zearalanone (ZAN), were not detected in 24 h and FMU samples [19]. If the co-exposure to both mycoestrogens is considered, 13% (*n* = 12) of participants presented urinary biomarkers for ZEN and AOH in 24 h urine samples. This co-exposure was determined for only 5% (*n* = 5) of the participants when analysing the FMU samples (data not shown).

#### 2.1.3. Food Consumption Data

As presented in Table 2, the food category with the highest reported consumption was “non-alcoholic drinks”, mainly due to the consumption of water (data not shown). Regarding the remaining food groups, “fruits and vegetables” was the group presenting the highest median consumption, followed by “cereals”, “dairy products”, “meat, fish and eggs”, and “cookies, biscuits, and sweets”. The consumption reported for all food categories did not present statistically significant differences between the first and the second interview (*p* > 0.05).

### 2.2. Link Between Food Consumption and Exposure Levels to Mycotoxins

The results presented in Table 3 summarize the statistical links between the consumption of some food items and the ZEN and AOH urinary levels of biomarkers. The Generalized Linear Models (GLM) considered the log-transformed urinary biomarkers as dependent variables, and the food consumption data of the second interview as independent variables. 

Regarding ZEN, three different models were obtained for results of ZEN’s urinary biomarkers. The meat consumption was positively associated with urinary levels of ZEN-14-GlcA and total ZEN. The association between consumption of meat and exposure to ZEN was previously reported by Bandera et al. (2011) in the Jersey Girls Study [21]. When considering the results obtained for FMU samples, it was possible to develop only one model; the consumption of cheese and fresh-cheese was positively associated with urinary levels of ZEN. Despite the lactational transfer of ZEN being considered low [42], the occurrence of ZEN in milk was reported in some studies showing evidence of possible carry-over [43,44]. Humans can be indirectly exposed to ZEN through consumption of animal products that have themselves been exposed [45], and these results are corroborated by previous reports of feed contamination in Portugal. Almeida et al. [46] reported 45% of feed samples (cows, ewes, goats) contaminated with ZEN, in low levels and below the recommended value of 500 µg/kg, and if considering specifically feed for cows the percentage of positive samples reported was 54.4% [46]. In a more recent review, Abrunhosa et al. reported a total of 25% of feed samples were contaminated by ZEN [47]. Cereal-based foods are well recognized as important determinants of exposure to ZEN and are thus regulated regarding the occurrence of ZEN [29]. Nevertheless, the need for a deeper knowledge on the occurrence of ZEN in foods from animal origins is also recognized by EFSA and Agence nationale de sécurité sanitaire de l’alimentation, de l’environnement et du travail (ANSES) (former Agence française de sécurité sanitaire des aliments (AFSSA)) in several reports [28,48,49] and should be properly addressed for a better risk estimation. Data generated under the present study, where animal products were found to be important contributors for the human exposure to ZEN, corroborate the importance of an extended assessment of the presence of ZEN in these products.

Positive and significant associations were found between the consumption of breakfast cereals and AOH urinary levels in 24 h urine samples. These results are consistent with the occurrence data reported so far for AOH. Cereals and cereal-products are one of the food commodities often contaminated with *Alternaria* toxins, including AOH [36,50]. The model obtained for first morning urines and food consumption revealed a positive association for meat and sweets. There are no reported data for the occurrence of AOH in animal products such as meat; on the contrary, there are available data reporting the occurrence of AOH in sweets [36]. Nevertheless, results obtained for this model should be considered carefully due to the lack of support from occurrence data. The food group of vegetables and fruits, which is frequently reported as being contaminated with *Alternaria* toxins, was not found as a determinant for the urinary levels of AOH [34,36].

### 2.3. Estimation of Exposure of Portuguese Population by Sex, Age Group and Region to Mycoestrogens

Data collected under the IAN-AF and the models developed in the present study (detailed in Section 2.2) allowed the estimation of exposure to mycoestrogens for a representative sample of the Portuguese population (*n* = 5811) stratified by region, sex, and age groups. 

The usual exposure for the 5811 participants was estimated using SPADE software (Statistical Program to Assess Dietary Exposure, R package SPADE.RIVM), a tool developed by RIVM [51]. Results are presented in Table 4. 

Regarding ZEN, the median estimate of PDI applying modelling was 0.240 µg/kg bw/day. The estimation of the percentage of participants that would exceed the tolerable daily intake for total ZEN (38.6%) was similar to the percentage determined by Martins et al. (24%) [19], with considerably lower estimates of intake for the high percentiles of exposure [19]. Regarding AOH, the median estimate for PDI applying modelling was 0.253 µg/kg bw/day. As there are no reference values for the intake, it is not possible to compare this exposure to a reference value [36]. A recent exposure assessment performed by EFSA [32] using occurrence and consumption data estimated an overall lower intake for AOH [32].

Results for the estimated exposure of 5811 participants, stratified by sex, age, and region, and the percentage of participants from each category that exceeded the TDI established for ZEN are presented in Table 5.

Regarding sex, males presented the highest exposure for ZEN and AOH, and this pattern was obtained not only for the estimated exposure (urinary levels), but also for the estimated PDI where the body weight and urinary volume were also considered. With the exception of exposure to AOH (urinary levels), all the exposure parameters presented statistically significant differences between males and females (*p* < 0.05). Regarding the exposure to ZEN, it was estimated that 22.5% and 15.0% of males and females, respectively, may exceed the TDI of 0.250 µg/kg bw/day. Regarding age categories, children and adolescents presented higher estimates than the remaining age groups for exposure to AOH. These results are probably due to higher consumption of breakfast cereals (data not shown) than other age groups, and to a lower proportion body weight/food consumption. For ZEN, children and adolescents did not present in general the highest estimates for exposure and PDI; however, the children age group was where a higher percentage of participants exceeded the TDI (58.2). Recently, Gratz et al. [52] estimated through a human biomonitoring study that 5% of the participants (children 2–6 years) may exceed the TDI for ZEN. Regarding the geographical distribution of exposure, and although all estimates for exposure and PDI presented statistically significant differences among the different considered categories, the estimated results followed a similar pattern for the seven regions of Portugal. The estimated PDI to AOH was higher for Algarve and Madeira. Regarding exposure to ZEN, Algarve and Lisbon Metropolitan Area presented the highest PDI estimates. This pattern of exposure, with relevant percentages of population exceeding the TDI, raises a potential health concern due to the health effects attributed to ZEN exposure, such as liver toxicity, reproductive toxicity, genotoxicity, and immunotoxicity [53].

Results obtained under this study are the first estimates of exposure to AOH and ZEN for a representative sampling of the Portuguese population. For ZEN, and since there is an established TDI, it was possible to estimate the percentage of participants that may exceed the reference intake value, and whose exposure could potentially represent a health concern. 

This study gathered data from different datasets: dataset of IAN-AF of 5811 participants (food consumption and sociodemographic data) and dataset of IAN-AF of 94 participants (food consumption, sociodemographic data, and paired urine samples), complemented with the dataset obtained by Martins et al. [19]. Data used in this study for modelling was obtained at the individual level (food consumption data, mycotoxin’s urinary biomarkers, body weight) and included urine samples collected through a standardized protocol for the entire survey. The sample collection was performed in parallel with the second interview, thus contributing for the quality of estimated data. 

Nevertheless, the interpretation of these estimates should be considered carefully since they are also affected by a degree of uncertainty. Left-censored data of urinary biomarkers was replaced by a multiple imputation method which keeps variability in the low levels of distribution but not exemption from uncertainty. The estimations of exposure and PDI are based on modelled data, and a fixed value for daily urinary volume was assumed (48 mL/kg for participants ≤ 5 years, 36 mL/kg for participants > 5 years and ≤ 11 years, and 24 mL/kg for participants ≥12 years) [54]. Additionally, these estimates considered only the food consumption variables that remained significant in the statistical models, leaving aside other possible sources of exposure. In this specific study, the generated models considered ZEN exposure through meat consumption, however, other food groups, e.g., cereal-based products, are traditionally considered as the main sources of ZEN exposure. This fact supports the need to consider a future review of legislation and for example inclusion of animal products as a possible source of ZEN exposure. Even considering the uncertainty associated with modelling and results obtained with this approach, data presented herewith indicates for the first time a potential health concern for the Portuguese population since a percentage of participants (38.6%) are estimated to surpass the tolerable daily intake for ZEN. The over-exposure of children is again demonstrated when compared to other age groups, meaning that this is an issue requiring further assessments. It should be reinforced that ZEN is considered more potent than BPA, one of the endocrine disruptors that raise more concern [13,14]. 

Despite the uncertainties referred, these results are important contributors in a public health perspective. They highlight the importance of properly and periodically assessing the exposure of the Portuguese population to mycotoxins, with the development of epidemiological studies including collection of blood paired with urine samples for a broader view on exposure, and consequently a more accurate risk characterization. These assessments will make possible the continuous identification of vulnerable population groups and the evaluation of time trends regarding exposure. If needed, and using the precautionary principle, the implementation of control strategies for the contamination levels of food products should be put in place, as well as the establishment of health-based guidance values for intake for emerging mycotoxins as AOH [55].

## 3. Conclusions

The estrogenic effects of ZEN and AOH represent a potential threat from public health and economic perspectives. Through mathematical modelling of HBM and food consumption data, it was possible to estimate the exposure of the Portuguese population to ZEN and AOH for a representative sampling of the Portuguese population stratified by age, sex, and region. These estimates revealed that the Portuguese population is exposed to ZEN in concentrations that are very close to the tolerable daily intake, and to AOH in concentrations higher than the ones determined in a previous study. There is also a contribution for a deeper knowledge of the potential exposure to endocrine disruptors in Portugal, with more data generated for these two mycoestrogens.

The importance of the development of biomonitoring studies linked with food and health surveys is highlighted in this study, since a more complete analysis has become possible. The acquisition of data from participants in these three domains opens the possibility of designing tailored public health interventions aiming to reduce exposure levels and the potential associated toxic effects.

## 4. Materials and Methods 

### 4.1. Participants

For the National Food, Nutrition, and Physical Activity Survey (IAN-AF), sampling was performed in two stages: first, based on the random selection of primary health care units, stratified by the seven Nomenclature of Territorial Units for Statistics (NUTS II; weighted by the number of individuals registered in each health unit); and second, based on the random selection of registered individuals in each health unit, according to sex and age groups [40]. From these, a convenience sample of 94 participants was recruited to participate in the biological sample collection for human biomonitoring studies. First morning urines and 24 h urine paired samples were collected on the previous and the day itself of the second interview and following a standardized protocol, in the conditions previously described by Martins et al. [19]. Ethical approval was obtained from the National Commission for Data Protection (Authorization number 4940/2015) and the Ethical Committee of the Institute of Public Health of the University of Porto (Decision number CE). All participants provided their written informed consent according to the Ethical Principles for Medical Research involving human subjects expressed in the Declaration of Helsinki and the national legislation. Data collection was performed under pseudo-anonymization, and all documents with identification data were treated and stored in a different dataset [40]. 

Considering the sampling strategy presented above, in the present study two groups of participants were considered. The first group, used to model food consumption and exposure to ZEN and AOH, included the 94 participants from whom we had HBM data (obtained through measurement of mycotoxins in urine samples as reported by Martins et al. [19]) and reported consumption data. The second group, for whom we estimated their exposure to ZEN and AOH using the modelling tools generated in this study, includes 5811 participants that reported consumption data.

### 4.2. Food and Sociodemographic Questionnaires

Participants performed two non-consecutive 24 h recalls, 8–15 days apart from each other, and an attempt was made to schedule the second interview for a day different from the first interview (*n* = 5811). The interview-based dietary assessment performed using computer-assisted personal interview (CAPI) (eAT24 software, SilicoLife, Braga, Portugal) allowed us to obtain a detailed description and quantification of foods, recipes, and food supplements consumed in the course of the preceding day. All foods, including beverages and composite dishes/recipes consumed during the previous 24 h period, were quantified as eaten. Several methods were used to assist participants in quantifying the food consumption such as: photo method, household measure method, weight or volume method, and standard unit method. Food categories comprised three levels of aggregation [40,56]. For the present study, seven food categories in the 1st and 2nd levels of aggregation were considered for the modelling approach: “fruits and vegetables” (fruits, vegetables, pulses, nuts, and oilseeds), “dairy products” (milk, cheese, yoghurt, milk cream), “cereals” (pasta, rice and other grains, flours and bakery powders, breakfast cereals and bars), “meat, fish, and eggs”(meat, fish, eggs), “cookies, biscuits, and sweets” (sweets, cakes, cookies, and biscuits), “non-alcoholic drinks” (tea, coffee, and water), and “alcoholic drinks” (wine, beer, and other drinks).

The questionnaires included sociodemographic data. Sex and age (calculated using the first interview date and birth date) were automatically imported from datasets obtained from the National Health Registries and checked during the first contact with the participants. Information on marital status, number of completed years of education, professional situation, household structure, and household monthly income was collected in a format of closed questions [40,56].

### 4.3. Exposure Data to ZEN and AOH Using HBM Data

ZEN and AOH urinary biomarkers were used to estimate the exposure of the Portuguese population, taking into account the results obtained by Martins et al. [19]. Data regarding urinary biomarkers were obtained for 24 h urine and first morning urine paired samples using a QuEChERS-based procedure (Quick, Easy, Cheap, Effective, Rugged, Safe) for sample preparation followed by identification and quantification by liquid chromatography with mass spectrometry detection (LC-MS/MS). The analytical method was previously optimized by Vidal et al. [57] and is described in detail by Martins et al. [19].

The probable daily intake (PDI) was estimated considering the following excretion rates: 9.4% for ZEN [15] and 8.3% for AOH [30]. Regarding the left-censored data obtained for urinary biomarkers results, a multiple imputation procedure was applied based on 20 simulations and with a maximum of 100,000 for cases and parameters [19]. This procedure allowed us to keep variability within the results below the limit of detection (LOD) [58]. The complete dataset was used for the modelling approach 

### 4.4. Modelling Approach for the Food Consumption and HBM Data

Data obtained by Martins et al. [19] for urinary levels of ZEN, ZEN-14-GlcA, Total ZEN (Sum of ZEN, ZEN-14-GlcA and α-ZEL considering the mass ratio between the parent compound and the metabolites), and AOH, expressed as volume weighted concentrations (µg/L), creatinine (crea) adjusted concentrations (µg/g crea), and daily excretion (µg/day) were used for the modelling approach. These data were compared with food consumption data (1st and 2nd level of aggregation, in a total of 30 variables) obtained with food questionnaires. Both variables (biomarkers and food consumption) were compared as continuous variables by bivariate analysis (Spearman’s correlation coefficient) (*n* = 94). Considering that significant associations between food consumption of last 24 h and urinary biomarkers are expected for mycotoxins with short half-lives [39], only consumption data from the second interview was considered for this modelling. 

Food consumption variables associated with urinary biomarkers concentration (*p* < 0.2) were retained for the multivariate analysis. For the multivariate analysis, the Generalized Linear Model was chosen due to the non-normality of urinary biomarkers’ distributions. For the model, food consumption variables were considered as independent variables, and urinary biomarkers levels were considered as dependent variables. Three types of Generalized Linear Model (GLM) were tested i) linear distribution; ii) gamma distribution; and iii) linear distribution with dependent variable log transformed. Variables were retained and considered to contribute significantly to the GLM if *p* < 0.1. The criteria considered for assessing the adjustment of models were the Spearman correlation coefficient and Omnibus test. Residuals analysis was performed. 

The models developed were used to derive HBM and PDI data for the group of 5811 participants of IAN-AF study. For estimation of usual exposure, the models were applied to consumption data of both interviews using SPADE software (Statistical Program to Assess Dietary Exposure, implemented in R software as package SPADE.RIVM) [51], and an overall analysis considering the weights for the Portuguese population was performed, presenting mean, median, and percentiles 75 and 95 for HBM (µg/g creatinine) and PDI (µg/kg bw/day). For estimation of exposure stratified by sex (male; female), age (children 0–9 years; adolescents 10–17 years; adults 18–64 years; elderly >64 years), and region (north, centre, Lisbon Metropolitan Area, Alentejo, Algarve, Madeira, Azores), a descriptive and inferential analysis was performed, presenting mean, median, and percentiles 75 and 95, and the results for Mann-Whitney and Kruskal Wallis non-parametric tests. 

The estimates of PDI were performed considering the derived HBM data and the individual body weight. For daily urinary volume the following values were considered: 48 mL/kg for participants ≤ 5 years, 36 mL/kg for participants > 5 years and ≤ 11 years, and 24 mL/kg for participants ≥ 12 years [54].

Normality of distributions was verified by Kolmogorov-Smirnov test. Statistical analysis was performed with SPSS v.24 (manufacturer, city, abbreviation of state (if it has), country) and R software.

## Figures and Tables

**Table 1:** Sociodemographic characteristics of sub-sample of participants in human biomonitoring (HBM) study (*n* = 94) and participants in National Food and Physical Activity Survey (IAN-AF) study (*n* = 5811) [40,41].

	Participants in HBM Study (*n* = 94)		Participants in IAN-AF Study (*n* = 5811)	
	*n*	%	*n*	%
**Sex**				
Male	48	51.1	2793	48.1
Female	46	48.9	3018	51.9
**Age**	-	-		
Children (0–9 years)	-	-	1327	22.8
Adolescents (10–17 years)	-	-	632	10.9
Adults (18–64 years)	81	86.2	3102	53.4
Elderly (>64 years)	13	13.8	750	12.9
**Region**				
North	74	78.7	989	17.0
Centre	20	21.3	1014	17.4
Lisbon Metropolitan Area	-	-	809	13.9
Alentejo	-	-	670	11.5
Algarve	-	-	766	13.2
Madeira	-	-	779	13.4
Azores	-	-	784	13.5
**Educational level**				
≤9 years	48	51.1	1530	26.3
10–12 years	25	26.6	2587	44.5
>12 years	21	22.3	1675	28.8
Do not know/answered	-	-	19	0.3
**Working condition**				
Worker for remuneration or profit	52	55.3	2119	55.0
Unemployed	14	14.9	444	11.5
Other ^1^	28	29.8	1286	33.4
Do not know/answered	-	-	3	0.1
**Household monthly income (€)**				
<485 €	9	9.6	362	9.4
485–970 €	22	23.4	1015	26.3
971–1455 €	30	31.9	875	22.7
1456–1940 €	16	17.0	514	13.3
More than 1941 €	13	13.8	708	18.4
Do not know/answered	4	4.3	378	9.8

^1^ retired, permanently disabled, student, domestic worker, performing military service or mandatory community service.

**Table 2:** Food consumption reported in edible grams per day (g/day) by the two groups of IAN-AF: *n* = 94 and *n* = 5811. [41].

	1st Interview (g/day)						2nd Interview (g/day)					
	*n* = 94			*n* = 5811			*n* = 94			*n* = 5811		
	Median	IQR	P95	Median	IQR	P95	Median	IQR	P95	Median	IQR	P95
Fruits and vegetables	297.4	186.2–528.7	699.1	272.6	159.4–408.0	662.7	311.9	177.9–465.8	783.9	263.4	153.7–393.2	655.4
Dairy products	193.0	27.6–384.7	744.8	272.8	118.9–462.1	794.7	222.8	100.9–326.3	528.4	268.8	109.0–460.1	789.7
Cereals	287.5	186.8–393.6	661.8	264.4	169.1–379.2	623.9	278.1	179.8–416.7	720.0	256.1	167.6–366.4	606.2
Meat, fish, and eggs	188.7	117.8–284.4	535.9	133.7	69.5–220.8	391.1	165.9	94.5–275.3	457.2	134.2	68.9–221.7	405.5
Cookies, biscuits, and sweets	39.6	10.4–121.8	209.3	29.0	6.0–101.2	240.0	38.9	9.5–102.5	259.4	28.0	6.0–100.0	242.4
Non-alcoholic drinks	1273.0	726.2–1822.8	2884.3	899.1	412.1–1551.0	2351.6	1183.1	742.0–1811.8	2548.7	866.1	410.0–1514.2	2329.4
Alcoholic drinks	8.3	0.0–251.1	921.9	0.0	0.0–27.7	582.2	7.6	0.0–238.4	979.9	0.0	0.0–25.3	591.3

IQR = Interquartile range; P95 = Percentile 95.

**Table 3:** Effect of consumption of food categories and the urinary levels of AOH, ZEN and ZEN-14-GlcA.

Mycotoxin	Urine Sample	Model	Urinary Biomarker	Food Category	Regression Coefficients	*p* Value	*R*	Omnibus
**ZEN**	24 h U	1	ZEN-14-GlcA (µg/g crea)	Meat	0.001	0.033	0.220	0.033
		2	Total ZEN (µg/g crea)	Meat	0.001	0.045	0.217	0.047
	FMU	3	ZEN (µg/g crea)	Cheese, Fresh-cheese	0.004	0.029	0.256	0.031
**AOH**	24 h U	4	AOH (µg/g crea)	Breakfast Cereals	0.010	0.019	0.153	0.021
		5	AOH (µg/day)	Breakfast Cereals	0.009	0.022	0.197	0.023
	FMU	6	AOH (µg/g crea)	Meat	0.001	0.020	0.285	0.011
				Sweets	0.002	0.003		

R = Spearman’ Correlation coefficient; Omnibus = Adjustment of model; AOH = Alternariol; ZEN = Zearalenone; ZEN-14-GlcA = Zearalenone-14-Glucuronide; Total ZEN = Sum of ZEN, ZEN-14-GlcA and α-ZEL considering the mass ration between the parent compound and the metabolites; FMU = First Morning Urine; 24 h U = 24 h urine.

**Table 4:** Estimated usual exposure to mycoestrogens of 5811 participants of IAN-AF, weighted for the Portuguese population distribution.

	Distribution					Reference Intake	
	*P25*	*Median*	*Mean*	*P75*	*P95*	*RVI*	*% ≥ RVI*
Total ZEN							
Exposure (µg/g crea)	0.587	0.648	0.655	0.714	0.823	-	-
PDI (µg/kg bw/day)	0.217	0.240	0.242	**0.265**	**0.305**	0.250	38.6%
AOH							
Exposure (µg/g crea)	0.322	0.582	0.621	0.742	1.053	-	-
PDI (µg/kg bw/day)	0.146	0.253	0.268	0.318	0.439	-	-

PDI = Probable Daily Intake; AOH = Alternariol; Total ZEN = Sum of ZEN, ZEN-14-GlcA and α-ZEL considering the mass ration between the parent compound and the metabolites; RVI = Reference Value for Intake, 0.250 µg/kg bw/day for ZEN (group TDI). Highlighted values reveal PDI > RVI. P25 = Percentile 25; P75 = Percentile 75; P95 = Percentile 95.

**Table 5:** Estimated exposure to mycoestrogens of 5811 participants of IAN-AF, stratified by sex, age, and region.

	Distribution Exposure (µg/g crea); PDI (µg/kg bw/day)				Reference Intake
	*Median*	*Mean*	*P75*	*P95*	*% ≥ RVI*
**AOH**					
Sex *					
Male	0.445; 0.127	0.873; 0.294	0.445; 0.254	2.270; 0.656	-
Female	0.445; 0.127	0.670; 0.230	0.445; 0.254	1.401; 0.581	-
Age **					
Children (0–9 years)	0.445; 0.254	0.651; 0.340	0.465; 0.254	1.339; 0.614	-
Adolescents (10–17 years)	0.445; 0.191	1.206; 0.396	1.339; 0.387	4.036; 1.349	-
Adults (18–64 years)	0.445; 0.127	0.779; 0.224	0.445; 0.127	1.529; 0.442	-
Elderly (>64 years)	0.445; 0.127	0.558; 0.160	0.445; 0.127	1.003; 0.290	-
Region **					
North	0.445; 0.127	0.696; 0.239	0.445; 0.254	1.339; 0.431	-
Centre	0.445; 0.127	0.631; 0.213	0.445; 0.254	1.339; 0.515	-
Lisbon Metropolitan Area	0.445; 0.127	0.828; 0.281	0.445; 0.254	2.270; 0.656	-
Alentejo	0.445; 0.127	0.674; 0.231	0.445; 0.254	1.367; 0.607	-
Algarve	0.445; 0.127	0.870;0.286	0.445; 0.254	2.270; 1.080	-
Madeira	0.445; 0.127	0.715; 0.249	0.445; 0.254	1.763; 0.619	-
Azores	0.445; 0.127	1.001; 0.343	0.586; 0.254	2.270; 0.952	-
**Total ZEN**					
Sex *					
Male	0.607; 0.185	0.682; 0.203	0.741; 0.250	1.107; 0.316	22.5
Female	0.567; 0.158	0.606; 0.180	0.644; 0.218	0.839; 0.290	15.0
Age **					
Children (0–9 years)	0.533; 0.255	0.561; 0.263	0.586; 0.280	0.728; 0.330	58.2
Adolescents (10–17 years)	0.622; 0.178	0.688; 0.194	0.741; 0.218	1.038; 0.308	13.6
Adults (18–64 years)	0.611; 0.153	0.678; 0.170	0.739; 0.185	1.065; 0.265	6.7
Elderly (>64 years)	0.558; 0.140	0.606; 0.152	0.646; 0.163	0.867; 0.218	2.4
Region **					
North	0.519; 0.175	0.643; 0.194	0.696; 0.250	0.989; 0.300	20.2
Centre	0.584; 0.168	0.636; 0.188	0.692; 0.225	0.918; 0.299	17.8
Lisbon Metropolitan Area	0.587; 0.173	0.653; 0.194	0.706; 0.250	1.024; 0.313	18.8
Alentejo	0.586; 0.173	0.649; 0.191	0.711; 0.245	1.032; 0.299	17.3
Algarve	0.586; 0.170	0.665; 0.196	0.694; 0.250	1.079; 0.315	19.3
Madeira	0.565; 0.160	0.613; 0.184	0.647; 0.223	0.871; 0.295	17.9
Azores	0.577; 0.170	0.643; 0.193	0.695; 0.250	0.959; 0.310	18.8

* Mann-Whitney test (*p* < 0.05); AOH exposure did not present significant differences regarding sex; **: Kruskal-Wallis test (*p* < 0.05); significant differences were found for all the categories of age and region, and for exposure and probable daily intake predicted. Reference Value for Intake (RVI) = 0.250 µg/kg bw/day for ZEN (group TDI).
